# Medical Opinion and Movement

**Published:** 1907-02-02

**Authors:** 


### MEDICAL OPINION AND MOVEMENT.

A DEPUTATION from the Infants' Health Society, including among its members Sir Thomas Barlow, Sir Lauder Brunton, and Mr. Mayo Robson, waited upon the President of the Local Government Board, and tendered him some valuable advice in view of the proposed Bill to legalise the establishment of milk depots by local authorities for supplying sterilised milk for infants. They pointed out that in the process of sterilisation milk loses in a great measure its nutritive qualities, and children fed on milk so treated were liable to develop scurvy and rickets, and were not entirely free from the dangers of zymotic diarrhœa. Recent reports from medical officers who have carefully investigated the matter have shown that the contamination of milk takes place largely in its transit from the source of supply to the consumer. What is most urgently needed is a thorough supervision of the handling of the milk right from the cow onwards. The deputation advocated refrigeration as an alternative to sterilisation. We understand that a joint committee of the National League for Physical Improvement, the National Health Society, and the Infants' Health Society, will shortly lay before the Local Government Board its practical conclusions on this question.

SIR A. E. WRIGHT's work on opsonins, and the consequent means of estimating the individual's power of reaction to the tubercle poison by taking the opsonic index, have given a great impulse to a more extensive treatment of tuberculous conditions by the injection of tuberculins. The "new" tuberculin, or tuberculin T.R., of Koch is generally used for this purpose. At University College Hospital, in several cases of tuberculosis requiring surgical treatment, injections of tuberculin have been made both before and after operation. The injections were controlled by estimation of the opsonic index, which was notably raised by them, and the excellent results which followed operation in each case suggest that a favourable influence was exerted upon the patients by these injections. Dr. H. D. McCulloch, of Bournemouth, has recently shown that the curative effect of the $x$-rays upon local manifestations of tuberculosis is also accompanied by increase in the opsonic index of the patient under treatment. He is of opinion that the therapeutic effect of the $x$-rays depends upon the induction of an auto-vaccination. The resolvent action of the $x$-rays on the encapsulating tissues about the tuberculous glands renders the vaccine accessible to the blood-stream. The applications of the $x$-rays were not accompanied by negative phases. This would suggest that the supposed vaccine liberated from the focus of disease by the $x$-rays differs in nature from the tuberculins prepared in the laboratory.

THERE is apparently an irresistible fascination to certain medical men to make themselves heard in the lay press and to pose as champions of the public weal, even at the risk of exciting prejudice against the profession to which they belong. An indictment against the profession such as Dr. James A. Rigby has recently made in the *Independent Review*, is much to be regretted, as it can only have the effect of diminishing the confidence placed by the public in the profession, and, further, is wholly unwarranted by facts. In an article entitled "The Surgeon's Power of Life and Death," Dr. Rigby tells his readers that illegitimate and indefensible operations are undertaken nowadays with light-hearted irresponsibility, and that the issues of life and death are placed in the hands of inexperienced young men "quite callous and indifferent to the true welfare of their patients, whom they look upon merely in the light of subjects to be experimented and operated upon." There is a great deal more in the same strain, but the few words we have quoted are sufficient to show the nature of the allegations and the seriousness of the charges he brings against the profession. If Dr. Rigby or his colleagues are in the habit of calling in inexperienced and callous youths to operate upon patients we can only express surprise. So far as our experience goes, the scope for operative efforts by the younger members of the profession is very limited, and their work is not only jealously watched over by the more skilled veterans, but the public themselves display a very discriminating partiality for the surgeon of mature experience and settled convictions.

IT is always interesting and instructive to know "how others see us," and especially pleasing to our vanity when the view is favourable. M. Montreuil, Director of the Salpêtrière, has recently paid a visit to the London hospitals and infirmaries, and his report to the Directeur de l'Administration Générale de l'Assistance Publique à Paris, has appeared in *Le Progrès Médical*. He finds much to praise and little to criticise in our methods and arrangements. He is deeply impressed above all else with the extreme cleanliness and orderliness that are displayed in every department of our institutions. He remarks that "This exquisite cleanliness is rendered possible by the habits and customs of the English people. The doctors are careful not to dirty the floors, and even the patients themselves show a consideration to which one is not accustomed in France." As he passes from out-patients to the wards, and thence to the operating theatre, he is continually filled anew with admiration for the cleanliness and well-being which prevail everywhere. He comments especially upon the decorations of the wards, the tables of flowers, the canaries in their cages, and the various other attempts to produce a bright and pleasing appearance. In fact, the whole report might be described as a song of praise of the cheerfulness and cleanliness M. Montreuil finds pervading all our public institutions. In commenting upon our fever hospitals, he expresses surprise that the linen was brought from the scarlet fever and diphtheria wards, and, without any precaution against infection, was taken by the ordinary route to the receiving room of the laundry; also that he himself was not asked to take any precaution either on entering or leaving the hospital.

